# Sex differences in the association between the uric acid to high density lipoprotein cholesterol ratio and mild cognitive impairment in patients with type 2 diabetes mellitus

**DOI:** 10.3389/fnut.2025.1667948

**Published:** 2025-10-17

**Authors:** Yafen Chu, Li Wang, Qiannan Guo, Yu Chang, Ni Lu, Qingyue Gao, Zhibang Song, Linlin Xu, Jumei Wang, Yan Chen, Li Ding, Bing Song

**Affiliations:** ^1^Department of Endocrinology, Ningbo Medical Center Lihuili Hospital, Ningbo, China; ^2^Department of Endocrinology, Anhui Provincial Hospital Affiliated to Bengbu Medical University, Hefei, China; ^3^Department of Endocrinology, The First Affiliated Hospital of USTC, Division of Life Sciences and Medicine, University of Science and Technology of China, Hefei, China; ^4^Department of Endocrinology, The First Affiliated Hospital of Jinzhou Medical University, Jinzhou, China; ^5^Department of Endocrinology, Ningbo No.2 Hospital, Ningbo, China; ^6^Department of Nephrology, The Affiliated Taizhou People's Hospital of Nanjing Medical University, Taizhou, China; ^7^Department of Endocrinology, Taikang Xianlin Drum Tower Hospital, Affiliated Hospital of Medical School, Nanjing University, Nanjing, China

**Keywords:** uric acid to high density lipoprotein cholesterol ratio, mild cognitive impairment, type 2 diabetes mellitus, nutrient metabolism disorder, sex difference

## Abstract

**Aim:**

Uric acid to high density lipoprotein cholesterol ratio (UA/HDL-c) related to nutrient metabolism disorder is associated with the onset of diabetic complications including mild cognitive impairment (MCI). However, the relationship between UA/HDL-c and MCI in type 2 diabetes mellitus (T2DM) patients with different gender remains unclear. Therefore, this study aims to explore the association between UA/HDL-c and MCI in female and male patients with T2DM.

**Methods:**

A total of 223 patients were stratified into either the control or the MCI group based on the presence or absence of MCI. Comparative analyses of clinical parameters were conducted, and the associations between UA/HDL-c and cognitive function were assessed across all patients as well as within female and male subgroups. Binary logistic regression was employed to identify independent risk factors for MCI in female and male patients with T2DM.

**Results:**

Compared to the 137 participants without MCI, the 86 individuals with MCI exhibited significantly higher levels of UA/HDL-c. Higher UA/HDL-c levels were associated with lower scores on the Montreal Cognitive Assessment, which reflects global cognitive function, as well as with poorer performance on the Verbal Fluency Test and the Clock Drawing Test, which reflect executive and visuospatial functions in female patients, respectively. These associations were not observed in male patients. Furthermore, binary logistic regression analysis indicated that elevated UA/HDL-c levels were a risk factor for MCI in women, regardless of adjustments for age, duration of diabetes mellitus, and duration of hypertension.

**Conclusion:**

Elevated UA/HDL-c levels are not only associated with overall cognitive function in female patients with T2DM, but also specifically linked to impairments in executive function and visuospatial abilities. However, this association is not observed in male patients. Among women with T2DM, elevated UA/HDL-c levels serve as an independent risk factor for the development of MCI. These findings suggest a sex-specific relationship between UA/HDL-c levels and cognitive dysfunction.

## 1 Introduction

The global incidence of type 2 diabetes mellitus (T2DM) has been steadily increasing. As reported by the International Diabetes Federation in 2021, the global diabetic population has exceeded 530 million individuals, with type 2 diabetes accounting for the vast majority of cases. China ranks among the countries with the highest burden, with national surveillance on chronic diseases and nutrition indicating that approximately 12.8% of adults aged 18 and above are affected. These figures highlight the substantial public health implications associated with the growing prevalence of T2DM (1). T2DM is a long-standing metabolic condition marked by sustained elevations in blood glucose levels and an inadequate compensatory insulin response (2). However, its manifestations extend beyond chronic hyperglycemia, encompassing a range of nutrition metabolic disturbances, particularly those involving lipid metabolism (3) and uric acid regulation (4, 5). As the prevalence of diabetes continues to rise, complications associated with the disease, including mild cognitive impairment (MCI), have garnered increasing attention from the public (6).

Factors associated with metabolic disturbances, such as prolonged chronic hyperglycemia (7, 8) and dysregulations in lipid metabolism (9–11) (including triglycerides, free fatty acids, and cholesterol), as well as uric acid (UA) metabolism (12), not only contribute to the onset of diabetes but are also closely linked to the development of its complications. Previous studies have shown that cholesterol metabolism, particularly the metabolism of high-density lipoprotein cholesterol (HDL-c), is not only associated with common diabetes complications but also with cognitive dysfunction in individuals with diabetes (13). Similarly, previous studies have identified a link between UA metabolism and MCI in individuals with T2DM (14). Moreover, one study suggests that HDL-c may mediate the effects of uric acid on diseases (15). Taking all of the aforementioned factors into account, we hypothesize that both HDL-c and UA may play roles in the development of diabetes-related cognitive dysfunction. Previous studies have indeed suggested that the UA/HDL-c not only indicates disruptions in HDL-c metabolism but also reflects abnormalities in UA metabolism, which are linked to various diabetic complications. Additionally, a research has identified a correlation between the UA/HDL-c and chronic kidney disease in Chinese patients with T2DM (16). Additionally, studies have indicated that individuals with diabetic nephropathy are more likely to experience MCI (17). In fact, a recent cross-sectional study has corroborated this finding (18).

Building on the above hypothesis, we propose that UA/HDL-c levels may be linked to the onset of MCI in individuals with T2DM. There are sex-based differences in the onset and progression of many diseases (19). Previous studies have highlighted that gender differences not only play a significant role in the progression of diabetes (20) but may also influence the occurrence and development of its complications (21). Therefore, we aim to further investigate the relationship between UA/HDL-c and MCI in patients with T2DM of different genders, including its association with specific aspects of cognitive impairment details through our present study.

## 2 Methods

### 2.1 Study design and ethical approval

A cohort of 233 individuals with a diagnosis of T2DM was enrolled from the Endocrinology Department of the First Affiliated Hospital of USTC. Among these, 86 participants with MCI were categorized into the MCI group, and the remaining 137 participants with intact cognitive function were included in the control group. Before enrollment, detailed explanations regarding the objectives and procedures of the study were provided to all participants, who then signed written informed consent forms. The research protocol received approval from the Ethics Committee at the First Affiliated Hospital of the USTC (Approval No.: 2023-RE-292).

### 2.2 Inclusion and exclusion criteria

Every individual included in the study received a diagnosis of diabetes according to the 1999 criteria established by the World Health Organization (22) and had a history of the condition lasting over 3 years. Among the enrolled subjects, 86 were diagnosed with MCI following the standards set by the MCI Working Group of the European Consortium on Alzheimer’s Disease (23). The exclusion criteria applied were consistent with those used in a previous study (24).

### 2.3 Clinical data

In our study, we collected patient information including age, sex, duration of diabetes mellitus (DMM), and duration of hypertension (DH) (recorded as 0 years for patients without a history of hypertension), high and body weight. The body mass index (BMI) was derived by dividing an individual’s weight in kilograms by the square of their height measured in meters. Laboratory data included glycated hemoglobin (HbA1c), triglycerides (TG), total cholesterol (TC), HDL-c, low-density lipoprotein cholesterol (LDL-c), UA, serum creatinine (Cr), and blood urea nitrogen (BUN). Based on the levels of UA and HDL-c, we calculated the UA/HDL-c. Using serum creatinine and BUN, we calculated the BUN/Cr ratio and estimated glomerular filtration rate (eGFR). All data were obtained from the patients’ medical records.

### 2.4 Neurocognitive performance

Neurocognitive performance was evaluated using a battery of standardized assessments in accordance with previously published protocols (25). Global cognitive function was quantified via the Montreal Cognitive Assessment (MoCA), with an additional point applied for individuals whose educational attainment was below 12 years, as per recommended correction criteria. Processing speed was examined using the Trail Making Test Part A (TMTA), while executive function was assessed through the Digit Span Test (DST), Verbal Fluency Test (VFT), and Trail Making Test Part B (TMTB), consistent with established methodologies. Immediate and delayed memory performance were assessed using the Auditory Verbal Learning Test—Immediate Recall (AVLT-IR) and Delayed Recall (AVLT-DR), respectively. Furthermore, contextual memory was measured using the Logical Memory Test (LMT).

### 2.5 Statistical methods

All statistical analyses were conducted using SPSS software (version 22.0; IBM Corp., USA). Continuous variables following a normal distribution—such as TC, LDL-C, and BUN—were described using mean values and standard deviations, with intergroup comparisons carried out via Student’s t-test. For non-normally distributed data, including age, DMM, DH, BMI, HbA1c, TG, HDL-C, UA, serum Cr, eGFR, and the UA/HDL-c, results were summarized as medians with interquartile ranges and analyzed using the Mann–Whitney U test. Categorical variables, such as sex, were expressed as counts and percentages, with group comparisons performed using the chi-square test. To assess the relationships among variables, Pearson and partial correlation analyses were applied, both before and after adjustment for confounders. Furthermore, binary logistic regression analysis was employed to identify independent risk factor of MCI.

## 3 Results

### 3.1 Comparison of clinical characteristics in T2DM patients with and without MCI

In this study, we conducted a comparative analysis of clinical parameters between T2DM patients with and without MCI. Although the design was cross-sectional and we did not perform strict matching for variables such as age, sex, DMM, or DH, no statistically significant differences were observed in these baseline characteristics between the two groups (all *p* > 0.05). Additionally, metabolic and renal function markers—including HbA1c, TG, TC, LDL-C, serum Cr, BUN, and eGFR—showed no significant variation (all *p* > 0.05). While a slight elevation in serum UA was noted in patients with MCI compared to those without, the difference did not reach statistical significance (*p* = 0.117). Interestingly, HDL-C levels were higher in the non-MCI group (*p* = 0.029). Most notably, the UA to HDL-C ratio was significantly elevated in T2DM patients with MCI (*p* < 0.001) (see Table 1).

**Table 1:** Comparation of clinical parameters and cognitive performance between Non-MCI group and MCI group.

	Non-MCI (*n* = 137)	MCI (*n* = 86)	*p*
Age (year)	62.00 (55.50, 71.00)	61.00 (55.75, 70.00)	0.719^a^
Female (*n*, %)	66, 48.18	34, 39.53	0.207^c^
DDM (year)	10.00 (6.00, 18.00)	10.00 (7.75, 17.00)	0.686^a^
DH (year)	0.00 (0.00, 10.00)	1.00 (0.00, 10.00)	0.350^a^
BMI (kg / m2)	24.22 (22.00, 26.02)	24.70 (22.66, 26.90)	0.641^a^
HbA1c (%)	7.60 (6.80, 8.70)	8.25 (7.00, 9.70)	0.107^a^
TG (mmol/L)	1.41 (0.97, 1.99)	1.55 (0.95, 2.33)	0.197^a^
TC (mmol/L)	4.46 ± 0.99	4.31 ± 1.01	0.310^b^
LDL-c (mmol/L)	2.65 ± 0.77	2.57 ± 0.79	0.446^b^
HDL-c (mmol/L)	1.12 (0.96, 1.29)	1.03 (0.87, 1.15)	0.029^a^
UA (umol/L)	301.20 (255.70, 344.35)	316.30 (270.55, 359.40)	0.117^a^
Cr (umol/L)	63.00 (55.00, 74.00)	64.00 (54.75, 76.50)	0.701^a^
BUN (mmol/L)	6.19 ± 1.60	6.46 ± 1.86	0.258^b^
BUN/Cr	25.31 (20.50, 28.48)	21.91 (16.75, 31.52)	0.289^a^
eGFR (ml/min/1.73 m^2^)	93.91 (86.30, 101.19)	91.21 (73.54, 101.14)	0.524^a^
UA/HDL-c	269.18 (211.65, 343.19)	309.51 (259.15, 388.89)	0.001^a^^*^
MoCA	28.00 (27.00, 29.00)	25.00 (23.00, 25.00)	<0.001^a^^*^
DST	12.00 (11.00, 15.00)	11.00 (8.00, 12.00)	0.002^a^^*^
VFT	17.00 (14.00, 20.50)	12.00 (9.00, 15.00)	<0.001^a^^*^
CDT	3.00 (2.00, 4.00)	2.00 (2.00, 3.00)	<0.001^a^^*^
TMTA	58.00 (47.00, 73.00)	72.00 (57.00, 83.00)	<0.001^a^^*^
TMTB	150.00 (116.00, 185.50)	183.00 (147.75, 215.25)	<0.001^a^^*^
AVLT-IR	17.00 (14.00, 21.00)	14.50 (11.00, 18.00)	0.049^a^^*^
AVLT-DR	8.00 (6.00, 9.00)	6.00 (5.00, 8.00)	<0.001^a^^*^
LMT	9.00 (6.00, 10.00)	7.00 (4.00, 9.00)	0.007^a^^*^

a The Mann–Whitney U test was employed for asymmetrically distributed variables.

b Student’s *t* test was employed for normally distributed variables.

c The Chi-square test was employed for categorical variables.

^*^p < 0.05. MCI, mild cognitive impairment; DDM, duration of diabetes mellitus; DH, Duration of hypertension; BMI, body mass index; HbA1c, glycosylated hemoglobin; TG, triglycerides; TC, total cholesterol; LDL-c, low density lipoprotein cholesterol; HDL-c, high density lipoprotein cholesterol; triglyceride glucose index, UA, uric acid; Cr, creatinine; BUN, blood urea nitrogen; BUN/Cr, blood urea nitrogen to creatinine ratio; eGFR, Estimated glomerular filtration rate; UA/HDL-c, uric acid to high density lipoprotein cholesterol ratio; MoCA, Montreal cognitive assessment; DST, digit span test; VFT, verbal fluency test; CDT, clock drawing test; TMTA, trail making test-A; TMTB, trail making test-B; AVLT-IR, auditory verbal learning test-immediate recall; AVLT-DR, auditory verbal learning test-delayed recall; LMT, logical memory test.

### 3.2 Comparison of neurocognitive performance in T2DM patients with and without MCI

To comprehensively evaluate cognitive impairment in patients with MCI, we compared not only the MoCA scores—reflecting global cognitive function—between T2DM patients with and without MCI, but also a range of domain-specific cognitive test scores. These included the DST, VFT, CDT, TMTA, TMTB, AVLT-IR, AVLT-DR scores and the LMT scores. Results indicated that T2DM patients with comorbid MCI exhibited a marked decline in MoCA scores (*p* < 0.001), suggesting global cognitive dysfunction. Furthermore, reductions in DST, VFT, and TMTB pointed to impairments in executive function; lower CDT scores and TMTA performance were indicative of visuospatial and processing speed deficits, respectively (all *p* < 0.05). Declines in AVLT-IR and AVLT-DR reflected impairments in immediate and delayed verbal memory, while decreased LMT scores suggested compromised contextual memory (all *p* < 0.05).

### 3.3 Association between UA/HDL-c and neurocognitive performance in patients with T2DM

To explore the relationship between serum HDL-c, UA, the UA/HDL-c, and neurocognitive function in patients with T2DM, Pearson correlation analyses were conducted. The findings revealed a statistically significant positive association between HDL-c levels and VFT scores (*R* = 0.168, *p* = 0.012), whereas UA levels were inversely correlated with MoCA (*R* = −0.171, *p* = 0.011) and CDT (*R* = −0.223, *p* = 0.001) scores. Moreover, the UA/HDL-c showed a positive correlation with MoCA (*R* = 0.169, p = 0.011) scores and a negative correlation with VFT (*R* = 0.137, *p* = 0.042) scores. These associations remained largely consistent in partial correlation analyses after adjusting for age, sex, DDM, and DH. Notably, although the association between the UA/HDL-c and VFT scores was no longer significant after adjustment, its correlation with MoCA scores persisted (*R* = −0.152, *p* = 0.024) (see Table 2).

**Table 2:** Partial association between UA/HDL-c (or HDL-c, or UA) and cognitive performance in patients with T2DM adjusting for age, gender, DDM, and DH.

	HDL-c		UA		UA/HDL-c	
	*R*	*p*	*R*	*p*	*R*	*p*
MoCA	0.113	0.094	−0.147	0.030^*^	−0.152	0.024^*^
DST	0.073	0.285	−0.071	0.295	−0.099	0.143
VFT	0.143	0.034 ^*^	−0.082	0.226	0.100	0.141
CDT	0.073	0.477	−0.223	0.001 ^*^	0.113	0.096
TMTA	−0.097	0.152	0.033	0.622	0.069	0.311
TMTB	−0.067	0.325	−0.048	0.477	−0.017	0.798
AVLT-IR	−0.099	0.144	−0.067	0.325	0.052	0.444
AVLT-DR	0.051	0.452	0.074	0.275	0.007	0.918
LMT	−0.039	0.567	−0.020	0.767	0.041	0.550

^*^p < 0.05. UA/HDL-c, uric acid to high density lipoprotein cholesterol ratio; HDL-c, high density lipoprotein cholesterol; UA, uric acid; T2DM, type 2 diabetes mellitus; DDM, duration of diabetes mellitus; DH, Duration of hypertension; MoCA, Montreal cognitive assessment; DST, digit span test; VFT, verbal fluency test; CDT, clock drawing test; TMTA, trail making test-A; TMTB, trail making test-B; AVLT-IR, auditory verbal learning test-immediate recall; AVLT-DR, auditory verbal learning test-delayed recall; LMT, logical memory test.

### 3.4 Difference of the relationship between UA/HDL-c and neurocognitive performance in female and male patients with T2DM

To further investigate the sex-specific differences in the association between the UA/HDL-c and neurocognitive performance, we conducted a subgroup analysis stratified by gender. The findings revealed pronounced disparities between male and female patients with T2DM. In female participants, the UA/HDL-c was significantly inversely correlated with multiple cognitive assessments, including the MoCA, DST, VFT, and CDT (all *p* < 0.05). In contrast, no such associations were observed in the male subgroup (all *p* > 0.05). Notably, after adjusting for age, DDM, and DH using partial correlation analysis, the inverse relationships between the UA/HDL-c and MoCA, VFT, and CDT scores remained significant in women (all *p* < 0.05) (see Tables 3, 4).

**Table 3:** Partial association between UA/HDL-c and cognitive performance in patients with T2DM adjusting for age, gender, DDM, and DH.

UA/HDL-c	Female		Male	
	*R*	*p*	*R*	*p*
MoCA	−0.209	0.040 ^*^	−0.094	0.309
DST	−0.159	0.119	−0.054	0.557
VFT	−0.246	0.015 ^*^	−0.019	0.836
CDT	−0.229	0.024 ^*^	−0.036	0.700
TMTA	0.063	0.541	0.068	0.462
TMTB	0.052	0.616	−0.076	0.411
AVLT-IR	−0.014	0.894	0.115	0.211
AVLT-DR	0.029	0.779	−0.018	0.847
LMT	−0.098	0.342	0.116	0.209

^*^p < 0.05. UA/HDL-c, uric acid to high density lipoprotein cholesterol ratio; T2DM, type 2 diabetes mellitus; DDM, duration of diabetes mellitus; DH, Duration of hypertension; MoCA, Montreal cognitive assessment; DST, digit span test; VFT, verbal fluency test; CDT, clock drawing test; TMTA, trail making test-A; TMTB, trail making test-B; AVLT-IR, auditory verbal learning test-immediate recall; AVLT-DR, auditory verbal learning test-delayed recall; LMT, logical memory test.

**Table 4:** Assessment of risk factors for MCI by binary logistic analysis in all patients adjusting for age, gender, DDM, and DH as well as in female and male patients with T2DM adjusting for age, DDM, and DH.

Gender	Risk factors	*β*	*p*	OR	95% CI	
					Lower	Upper
All	UA/HDL-c	0.004	0.004 ^*^	1.004	1.001	1.007
	Age	0.001	0.949	1.001	0.968	1.036
	Gender	0.090	0.766	1.094	0.604	1.981
	DM	0.011	0.592	1.011	0.971	1.053
	HBP	0.005	0.800	1.005	0.969	1.042
Female	UA/HDL-c	0.006	0.019 ^*^	1.006	1.001	1.012
	Age	0.044	0.116	1.045	0.989	1.104
	DM	0.004	0.910	1.004	0.942	1.069
	HBP	−0.007	0.802	0.993	0.943	1.046
Male	UA/HDL-c	0.002	0.176	1.002	0.999	1.005
	Age	−0.036	0.122	0.964	0.921	1.010
	DM	0.014	0.612	1.014	0.960	1.072
	HBP	0.010	0.721	1.010	0.957	1.066

^*^p < 0.05. MCI, mild cognitive impairment; T2DM, type 2 diabetes mellitus; DDM, duration of diabetes mellitus; DH, Duration of hypertension; UA/HDL-c, uric acid to high density lipoprotein cholesterol ratio.

### 3.5 Analysis for the risk factor for MCI in female and male patients with T2DM

To further investigate whether an elevated UA/HDL-c serves as an independent risk factor for MCI in individuals with T2DM, we conducted a binary logistic regression analysis. The findings revealed that, in female patients, a higher UA/HDL-c was one of the independent risk factors for MCI, irrespective of adjustments for age, DDM, and DH (OR = 1.007 and 1.006; *p* = 0.009 and 0.019). In contrast, among male patients, the UA/HDL-c was not independent risk factor for MCI, regardless of whether these covariates were accounted for (all *p* > 0.05).

## 4 Discussion

The increasing prevalence of diabetes is strongly associated with unhealthy lifestyle patterns and imbalanced dietary habits that contribute to nutrition metabolic disturbances (26). These metabolic abnormalities not only play a fundamental role in the pathogenesis and progression of diabetes itself but are also closely linked to its complications, including diabetes-related cognitive impairment (27). In this study, we examined and compared glycemic control, BMI, and lipid metabolism between T2DM patients with and without MCI. Although previous studies have suggested a possible association between elevated cholesterol levels and several single nucleotide polymorphisms in genes regulating lipid metabolism with cognitive decline, our findings did not reveal significant differences in total cholesterol or LDL-cholesterol between the two groups. However, HDL-cholesterol levels were notably higher in patients without cognitive impairment, which aligns with earlier observations indicating a potential protective role of HDL-c (13). Renal function has also been implicated in the pathophysiology of diabetic cognitive dysfunction (28), with diabetic nephropathy possibly contributing to cognitive decline (18). To explore this, we compared markers of kidney function—serum Cr and eGFR—between the 2 groups, but no significant differences were observed. Interestingly, we noted a mild elevation in serum uric acid levels among patients with MCI. While the difference did not reach statistical significance, it may suggest a potential association between altered uric acid metabolism and cognitive impairment. Elevated uric acid might reflect broader metabolic disruptions contributing to cognitive decline (29, 30), or alternatively, some studies propose that mildly increased uric acid could exert neuroprotective effects due to its antioxidant properties (14, 31).

Drawing on prior evidence linking HDL-c and serum UA with diabetes-related complications, including cognitive impairment, as well as our own findings showing differences in HDL-c and UA levels between T2DM patients with and without MCI, we further explored the associations between these biomarkers and cognitive performance. Our analyses demonstrated that both HDL-c and UA were significantly correlated with various neuropsychological test scores indicative of cognitive decline, regardless of adjustments for age, sex, DMM, and DH. Based on these observations, we hypothesized that the UA/HDL-c may serve as a more sensitive indicator of cognitive dysfunction. To test this, we conducted additional correlation analyses and found that the UA/HDL-c was consistently associated with scores on the MoCA, a global measure of cognitive function. This relationship persisted even after controlling for potential confounders, including age, sex, DMM, and DH. Although the correlation coefficient was modest (*R* = −0.152), the association remained statistically significant, suggesting that an elevated UA/HDL-c may represent an independent risk factor for MCI in T2DM patients. Indeed, in the overall population, elevated UA/HDL-c level was identified as an independent risk factor for the development of MCI in patients with T2DM, regardless of adjustments for age, sex, DM, and DH. These findings align with previous reports that underscore the close link between the UA/HDL-c and the development of diabetes (32).

As described above, UA/HDL-c demonstrated only a weak association with global cognitive performance, as measured by the MoCA score, in the overall patients. Additionally, prior evidence suggests that the pathogenesis of diabetes and its complications exhibits notable sex-based disparities (20, 21). Studies have shown that sex hormones can influence uric acid (33–35) and triglyceride metabolism (36, 37), suggesting that sex differences may contribute to variations in the UA/HDL-c ratio. In addition, other research indicates that sex hormones may potentially affect cognitive function (38, 39). This prompted us to investigate whether stratification by sex might reveal stronger associations within a specific subgroup. Notably, among female patients, a significant inverse correlation was observed between the UA/HDL-c and MoCA scores (Pearson’s *R* = −0.252). Even after adjusting for potential confounders such as age, DMM, and DH, the association remained statistically meaningful (*R* = −0.209). Furthermore, in women with T2DM, the UA/HDL-c also showed significant associations with measures of executive function (VFT scores) and visuospatial abilities (CDT scores), regardless of adjustment for age, DMM, and DH. These findings suggest that in female T2DM patients, the UA/HDL-c may be a sensitive marker not only for global cognitive status but also for specific cognitive domains, a pattern not observed in male counterparts. Further logistic regression analysis demonstrated that an elevated uric acid to UA/HDL-c independently increases the risk of MCI in female patients with T2DM, regardless of whether adjustments were made for age, duration of diabetes, or hypertension. These findings are consistent with prior reports indicating that a higher UA/HDL-c is linked to the development of diabetic complications, including diabetic retinopathy (40), diabetic kidney disease (41), diabetic peripheral neuropathy (42), abnormal bone mineral density (43), metabolic dysfunction-associated steatotic liver disease (44), cardiovascular disease (45), and may also contribute to neurological impairment (46), suggesting a potentially detrimental impact on women (47). Notably, a study had reported sex-specific differences in the relationship between UA/HDL-c and diabetes (48).

To the best of our knowledge, this is the first investigation to examine the association between the UA/HDL-c and MCI in individuals withT2DM. This study also provides a gender-stratified analysis, highlighting the relationship between UA/HDL-c and cognitive performance in male and female subgroups. Notably, our findings suggest that, among female patients, an elevated UA/HDL-c is significantly associated with impaired cognitive function and serves as an independent risk factor for MCI. Despite the novelty of these results, the study has certain limitations. Firstly, the sample size in our study is relatively limited. In our subsequent analyses, we performed subgroup analyses stratified by sex, which further reduced the number of participants in each group. Therefore, we must acknowledge that our study suffers from a relative insufficiency of sample size. Our study is a small-sample cross-sectional investigation, which, unlike case–control studies, did not include matching for age or sex. This may have introduced potential bias in the results. In the analysis of the overall cohort, we adjusted for both age and sex. In the subgroup analysis stratified by sex, we further adjusted for age. Although these adjustments and subgroup analyses may partially mitigate such bias, we acknowledge that this remains one of the limitations of our study. Furthermore, given the cross-sectional design, our findings can only demonstrate an association between the UA/HDL-C ratio and MCI, rather than a causal relationship. Secondly, in the present study, we also considered the potential relationship between hypertension and cognitive impairment. Although no significant difference in the duration was observed between patients with and without MCI, we nonetheless adjusted for hypertension duration in the subsequent analyses. Unfortunately, our dataset did not include detailed blood pressure information (such as systolic and diastolic values, antihypertensive medications, or ambulatory blood pressure monitoring results), which prevented us from conducting a more in-depth evaluation of its role in this study. We recognize this as one of the limitations of our work. Thirdly, regarding smoking and alcohol consumption, given their strong association with diabetic complications, patients with such histories were excluded at enrollment. Unfortunately, exposure to secondhand smoke is also relevant to diabetic complications; however, we lacked data on secondhand smoke exposure, which we recognize as a limitation of our study. Lastly, as a retrospective study, we did not have access to data on patients’ physical activity. With respect to medication use, we did collect information on the classes of drugs prescribed. However, detailed data on dosage and duration of treatment were unavailable, which restricted the scope of further analysis. Moreover, due to the limited sample size, certain medications were used by only a small number of patients, further constraining the possibility of subgroup analyses. We also regard this as one of the limitations of our study. Interestingly, our previous colleague evaluated the effects of different antidiabetic drugs on cognitive function by a recently published network meta-analysis (49).

## 5 Conclusion

Overall, higher UA/HDL-c appear to be independently associated with both global cognitive decline and specific deficits in executive and visuospatial functions among women with T2DM. In contrast, this correlation does not emerge in male counterparts. Notably, in female T2DM patients, an elevated UA/HDL-c constitutes a distinct risk factor for the onset of MCI. These results underscore a gender-specific link between UA/HDL-c balance and cognitive deterioration.

## Data Availability

The raw data supporting the conclusions of this article will be made available by the authors, without undue reservation.
