# Supplementary material for: Sex differences in the association between the uric acid to high density lipoprotein cholesterol ratio and mild cognitive impairment in patients with type 2 diabetes mellitus
Source: Front Nutr. 2025 Oct 17;12:1667948. doi: 10.3389/fnut.2025.1667948 (PMC12577417; doi:10.3389/fnut.2025.1667948)
Supplement: Supplementary file 1 [file Table_1.docx]

Supplementary - Table 1: Pearson association between UA/HDL-c (or HDL-c, or UA) and cognitive performance in patients with T2DM

|  | HDL-c | | UA | | UA/HDL-c | |
| --- | --- | --- | --- | --- | --- | --- |
|  | R | P | R | P | R | P |
| MoCA | 0.121 | 0.071 | -0.171 | 0.011 ^*^ | -0.169 | 0.011 ^*^ |
| DST | 0.085 | 0.203 | -0.099 | 0.141 | -0.117 | 0.081 |
| VFT | 0.168 | 0.012 ^*^ | -0.120 | 0.074 | 0.137 | 0.042 ^*^ |
| CDT | 0.049 | 0.463 | -0.223 | 0.001 ^*^ | 0.119 | 0.077 |
| TMTA | -0.089 | 0.185 | 0.034 | 0.614 | 0.063 | 0.350 |
| TMTB | -0.062 | 0.357 | -0.047 | 0.490 | -0.019 | 0.783 |
| AVLT-IR | -0.099 | 0.140 | -0.075 | 0.264 | 0.047 | 0.486 |
| AVLT-DR | 0.055 | 0.414 | 0.062 | 0.359 | -0.003 | 0.960 |
| LMT | -0.033 | 0.620 | -0.041 | 0.538 | 0.019 | 0.223 |

Notes: ^*^ P<0.05. Abbreviations: UA/HDL-c, uric acid to high density lipoprotein cholesterol ratio; HDL-c, high density lipoprotein cholesterol; UA, uric acid; T2DM, type 2 diabetes mellitus; DDM, duration of diabetes mellitus; DH, Duration of hypertension; MoCA, Montreal cognitive assessment; DST, digit span test; VFT, verbal fluency test; CDT, clock drawing test; TMTA, trail making test-A; TMTB, trail making test-B; AVLT-IR, auditory verbal learning test-immediate recall; AVLT-DR, auditory verbal learning test-delayed recall; LMT, logical memory test.

Supplementary-Table2: Pearson association between UA/HDL-c and cognitive performance in female and male patients with T2DM

| UA/HDL-c | Female | | Male | |
| --- | --- | --- | --- | --- |
|  | R | P | R | P |
| MoCA | -0.252 | 0.012 ^*^ | -0.108 | 0.236 |
| DST | -0.223 | 0.026 ^*^ | -0.073 | 0.421 |
| VFT | -0.297 | 0.003 ^*^ | -0.044 | 0.629 |
| CDT | -0.228 | 0.022 ^*^ | -0.041 | 0.653 |
| TMTA | 0.073 | 0.468 | 0.073 | 0.423 |
| TMTB | 0.049 | 0.631 | -0.037 | 0.687 |
| AVLT-IR | 0.036 | 0.719 | 0.078 | 0.394 |
| AVLT-DR | 0.054 | 0.591 | -0.011 | 0.903 |
| LMT | -0.095 | 0.345 | 0.136 | 0.135 |

Notes: ^*^ P<0.05. Abbreviations: UA/HDL-c, uric acid to high density lipoprotein cholesterol ratio; T2DM, type 2 diabetes mellitus; MoCA, Montreal cognitive assessment; DST, digit span test; VFT, verbal fluency test; CDT, clock drawing test; TMTA, trail making test-A; TMTB, trail making test-B; AVLT-IR, auditory verbal learning test-immediate recall; AVLT-DR, auditory verbal learning test-delayed recall; LMT, logical memory test.

Supplementary -Table 3: Assessment of risk factors for MCI by binary logistic analysis in all patients as well as in female and male patients with T2DM

| Gender | β | P | OR | 95% CI | |
| --- | --- | --- | --- | --- | --- |
|  |  |  |  | Lower | Upper |
| All | 0.004 | 0.002^*^ | 1.004 | 1.001 | 1.007 |
| Female | 0.007 | 0.009 ^*^ | 1.007 | 1.002 | 1.012 |
| Male | 0.003 | 0.085 | 1.003 | 1.000 | 1.006 |

Notes: ^*^ P<0.05. Abbreviations: MCI, mild cognitive impairment; T2DM, type 2 diabetes mellitus.
